# Epstein–Barr Virus and Human Endogenous Retrovirus in Japanese Patients with Autoimmune Demyelinating Disorders

**DOI:** 10.3390/ijms242417151

**Published:** 2023-12-05

**Authors:** Davide Cossu, Yuji Tomizawa, Leonardo Antonio Sechi, Nobutaka Hattori

**Affiliations:** 1 Department of Neurology, Juntendo University, Tokyo 1138431, Japan; zawa@juntendo.ac.jp (Y.T.); nhattori@juntendo.ac.jp (N.H.); 2 Biomedical Research Core Facilities, Juntendo University, Tokyo 1138431, Japan; 3 Department of Biomedical Sciences, Sassari University, 07100 Sassari, Italy; sechila@uniss.it; 4 Struttura Complessa di Microbiologia e Virologia, Azienda Ospedaliera Universitaria, 07100 Sassari, Italy; 5 Neurodegenerative Disorders Collaborative Laboratory, RIKEN Center for Brain Science, Saitama 3510918, Japan

**Keywords:** Epstein–Barr virus, HERV-W, multiple sclerosis, NMOSD, MOGAD

## Abstract

Multiple sclerosis (MS), neuromyelitis optica spectrum disorder (NMOSD), and myelin oligodendrocytes glycoprotein-antibody disease (MOGAD) are distinct autoimmune demyelinating disorders characterized by varying clinical and pathological characteristics. While the precise origins of these diseases remain elusive, a combination of genetic and environmental factors, including viral elements, have been suggested as potential contributors to their development. Our goal was to assess the occurrence of antibodies against pathogenic peptides associated with *Epstein–Barr* virus (EBV) and the human endogenous retrovirus-W (HERV-W) in serum samples obtained from Japanese individuals diagnosed with MS, NMOSD, and MOGAD and to make comparisons with a group of healthy controls (HCs). We conducted a retrospective analysis involving 114 Japanese participants, comprising individuals with MS (34), NMOSD (20), MOGAD (20), and HCs (40). These individuals were tested using a peptide-based enzyme-linked immunosorbent assay. A marked increase in antibody response against EBV nuclear antigen 1 (EBNA1)_386–405_ was observed in the serum of MS and MOGAD patients, as compared to HCs. Notably, we observed a correlation between antibodies against EBNA1_386–405_ and HERV-W_486–504_ peptides in a subset of the antibody-positive MS patients. These findings emphasize the involvement of EBV in the pathogenesis of MS and potentially MOGAD, suggesting its role in the reactivation of HERV-W.

## 1. Introduction

Autoimmune demyelinating disorders encompass a group of neurological conditions characterized by the immune system mistakenly attacking and damaging the myelin sheath, which serves as the protective covering of nerve fibers within the central nervous system (CNS) [1]. This assault on myelin can result in a wide spectrum of symptoms that depend on the location and extent of demyelination. Some of the most well-known autoimmune demyelinating disorders include multiple sclerosis (MS), neuromyelitis optica spectrum disorder (NMOSD), and myelin oligodendrocytes glycoprotein-antibody disease (MOGAD). Each of these conditions shares the hallmark feature of immune-mediated myelin damage, but they can exhibit distinct clinical characteristics and patterns of involvement within the CNS [2].

In the case of MS, the immune system primarily targets the myelin sheath in the CNS. This relentless attack leads to the formation of white matter lesions or the development of scar tissue within the nervous system [1]. These disruptions profoundly interfere with the normal transmission of nerve signals, causing a myriad of neurological symptoms and impairments. It is noteworthy that the worldwide prevalence of MS is estimated to be approximately 35.9 cases per 100,000 population [3]. However, there are pronounced geographical and ethnic variations in MS prevalence [3]. For instance, MS is more frequently diagnosed in individuals of European descent, particularly those of Northern European ancestry, in comparison to individuals of Asian, African, or Indigenous descent [3]. These differences in prevalence are believed to be influenced by genetic, environmental, and geographical factors, although the exact mechanisms remain a subject of ongoing research. Additionally, it is important to recognize the diverse clinical courses that MS can manifest. The primary categories include relapsing–remitting MS, which is characterized by episodic relapses and remissions; secondary progressive MS, marked by a gradual accumulation of disability after an initial relapsing course; and primary progressive MS, which entails a steady progression of disability from the onset without distinct relapses [4]. These clinical courses highlight the heterogeneity of MS and the need for tailored treatment approaches.

NMOSD constitutes a group of neuroinflammatory diseases of the CNS, primarily characterized by acute optic neuritis, transverse myelitis, and area postrema syndrome [5]. The prevalence of NMOSD varies, affecting between 0.5 and 10 individuals per 100,000 [6]. Notably, East Asians, including Japanese people, exhibit a higher prevalence of NMOSD, estimated at around 3.5 per 100,000, compared to individuals of White and other Asian racial groups [6]. Most patients diagnosed with NMOSD present IgG autoantibodies specific for the water channel aquaporin 4 (AQP4). These autoantibodies play a central role in activating the classical complement pathway, leading to an autoimmune astrocytopathy [7]. It is important to acknowledge that a subgroup of NMOSD patients is seronegative for AQP4 antibodies, highlighting an overlap in clinical features between NMOSD and MS [8]. Approximately 40% of NMOSD patients who lack AQP4 antibodies have been found to test positive for myelin oligodendrocyte glycoprotein (MOG) antibodies [9]. 

MOGAD is a relatively recent addition to the spectrum of demyelinating disorders. It can manifest with a wide array of neurological symptoms, including optic neuritis, transverse myelitis, and other CNS-related manifestations [10]. The prevalence of MOGAD is estimated at approximately 1.3 to 2.5 cases per 100,000 [11].

In all these disorders, the precise cause of the autoimmune response remains not fully understood. While specific genetic variations have been linked to an increased risk of developing these disorders [12,13], it is fundamental to recognize that possessing these genetic factors does not guarantee the development of the disease. Environmental factors also play a key role in disease onset and progression.

Some viral and bacterial infections have been associated with the onset of autoimmune demyelinating disorders [2]. Clinical studies conducted in various world populations, including our studies conducted in Italy and Japan, suggested that coinfection by viruses such as *Epstein–Barr* virus (EBV), human W-family endogenous retroviruses (HERV-W), and mycobacteria apparently leads to a synergistic effect that exacerbates the progression of neuroinflammation [14,15,16]. It is noteworthy that these studies were conducted retrospectively with cohorts of approximately 100 patients, analyzing the humoral and cell-mediated responses against specific pathogenic components in therapy-free patients with relapsing–remitting MS. Additionally, a limited group underwent evaluation for the presence of antibodies against EBNA1 and HERV-W after a 6-month IFN-β therapy period [14,15].

It has been hypothesized that the EBV, a globally widespread γ-herpes virus capable of establishing latent infection with periodic reactivation, contributes to the pathogenesis of MS, either directly by infecting B cells [17] or indirectly by interacting with other genetic and environmental factors, such as the activation of neuropathogenic human endogenous retroviruses [18]. In a recent study conducted on Japanese and Sardinian subjects, we identified antibodies showing cross-reactivity between EBNA1_386–405_ and a peptide belonging to *Mycobacterium avium paratuberculosis* heat shock protein 70 [19]. *M. paratuberculosis* is a bacterium endemic in the Sardinian Island, which has been proposed as a potential risk factor for MS in susceptible individuals, and its encephalitogenic potential in experimental autoimmune encephalomyelitis (EAE) has been demonstrated [19].

Studies have suggested that active EBV replication may play a pathogenic role in NMOSD [20], as elevated levels of IgG antibodies to the early antigen were detected in a cohort of Japanese patients with NMO compared to MS and healthy controls (HCs) [20]. Interestingly, EBV has also been linked to several autoimmune disease associated with NMOSD [21], such as myasthenia gravis [22], systemic lupus erythematosus [23], and Sjogren’s syndrome [24]. Various hypotheses surround this connection: EBV may induce molecular mimicry, where viral antigens resemble self-antigens, contributing to the development or exacerbation of the disease. Additionally, EBV can influence the immune system, potentially causing dysregulation, particularly in individuals predisposed to autoimmune diseases, thereby triggering or worsening the autoimmune response. Persistent infection of B cells by EBV can lead to the activation of autoreactive B cells, which may contribute to the production of autoantibodies observed in autoimmune diseases. Lastly, evidence supports the role of genetic factors in susceptibility to both EBV infection and the development of autoimmune diseases, with certain individuals having a genetic predisposition to a higher susceptibility to both EBV infection and autoimmune disorders [25].

In addition, cases of anti-MOG antibody-positive acute disseminated encephalomyelitis [26], a CNS demyelinating disorder, have been reported following infectious mononucleosis due to primary EBV infection [27]. These data indicate that EBV may play a role in the development of autoimmune diseases through mechanisms of immune tolerance disruption that include molecular mimicry, B-cell transformation, and modulation of B-cell trafficking [17]. 

Regarding HERV-W, which constitute a significant portion of the human genomic DNA, they have also been associated with the pathogenesis of MS [28]. In a new study conducted with a group of Sardinian patients with relapsing–remitting MS, a robust antibody response against the pathogenic HERV-W_486–504_ peptide was observed, particularly in patients in the acute phase [29]. These findings reinforce the connection between the pathogenic HERV-W protein and disease severity. 

Therefore, we have set forth two primary objectives: firstly, we aim to validate the data obtained by screening for the first time the presence of antibodies against HERV-W in Japanese patients with MS, NMOSD, and MOGAD. Concurrently, we will examine all patients for the presence of EBV to both confirm the significant role of this virus in neuroinflammatory processes and to determine if there is a correlation between EBV infection and the activation of HERV-W.

## 2. Results

### 2.1. Humoral Response to EBNA1_386–405_ Peptide in Patients with MS, MOGAD, NMOSD, and Healthy Controls

**Figure 1:**
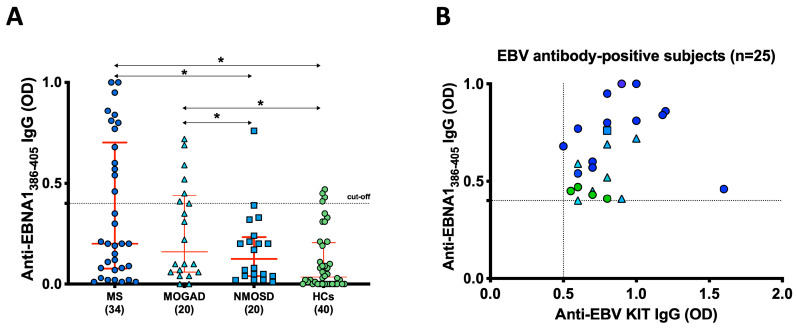
Humoral response and EBV EBNA1 analysis. (**A**) Humoral response against the EBNA_1386–405_ peptide observed in our study cohort. The red bars represent the median and interquartile range, and the results are displayed as the means of duplicate optical density (OD) values. (**B**) Correlation between the titers of EBNA1_386–405_ antibodies detected through peptide-based ELISA and EBNA1 antibodies detected via the EBV ELISA kit. Each shape and color in the figure corresponds to the titer of a single serum sample from a specific patient, as reported in graph A, with the dotted lines indicating the cutoff for positivity applied in each assay. (*) indicates statistically significant values.

In our study cohort, we observed a noteworthy humoral response against the EBNA1_386–405_ peptide (Figure 1A). Among the 34 MS patients, 13 individuals (38%; 95% confidence interval [CI]: 55–83%) exhibited a significant response (*p* = 0.04) in their serum. In the case of the 20 MOGAD patients, 7 of them (35%; [CI]: 55–83%) also showed a substantial response (*p* = 0.04). In contrast, only 1 out of the 20 NMOSD patients (5%; [CI]: 52–78%) demonstrated a similar response. This contrast was even more pronounced when compared to the 4 out of 40 HCs (10%) who exhibited the response (refer to Figure 1A). Notably, the peptide-based ELISA test displayed excellent reproducibility, with an inter-assay coefficient of variation (CV) of 8% and an intra-assay CV of 4%.

Among the EBNA1_386–405_-positive MS patients (*n* = 13), all were in a stable phase with an average expanded disability status scale (EDSS) score of 2. Of these, 8 were female and 5 were male. In the MOGAD group, 4 patients were female and 3 were male, with an average EDSS of 2.5.

To confirm the specificity of the antibodies targeting the epitope and validate the accuracy of our technique, we analyzed all EBNA1_386–405_-positive subjects (*n* = 25) using the EBV EBNA1 ELISA kit. Notably, all subjects exhibited double positivity in both the in-house peptide-based ELISA and the commercial ELISA assay (*p* < 0.001) (Figure 1B). 

In addition, we assessed the serum concentrations of EBNA1 using the EBV kit in EBNA1_386–405_-positive patients with MS, revealing a median value with an interquartile range of 55.2 (ranging from 45 to 80 AU/mL). Similarly, for the patients with MOGAD, the median was 55.2 (with an interquartile range of 36–66 AU/mL), while patients with NMOSD had a median value of 55.7. HCs displayed a median concentration of 40.9, with an interquartile range of 36–45 AU/mL.

### 2.2. Antibody Response to HERV-W_486–504_ Peptide in Patients with MS, MOGAD, NMOSD, and Healthy Controls

**Figure 2:**
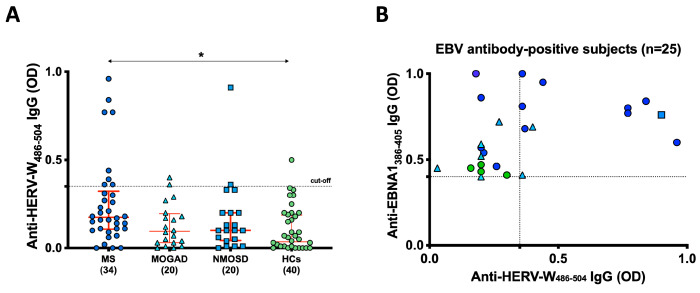
Antibody response to HERV-W_486–504_ peptide and association with EBNA1_386–405_ in MS, MOGAD, and NMOSD patients. (**A**) Presence of antibodies against the HERV-W_486–504_ peptide within our study cohort. The red bars signify the median and interquartile range, and the results are represented as the means of duplicate OD values. (**B**) Correlation between the titers of EBNA1_386–405_ antibodies and HERV-W_486–504_ antibodies. Each shape and color within the figure corresponds to the titer of a single serum sample from a specific patient, as reported in graph A, with the dotted lines indicating the cutoff for positivity applied in each assay. (*) indicates statistically significant values.

In our study, we examined all subjects for the presence of antibodies against the HERV-W_486–504_ peptide (Figure 2A). We detected a significant immune response (*p* = 0.03) against this epitope in 8 out of 34 patients with MS (23%; [CI]: 65–86%), which was notably higher when compared to the 2.5% of HCs who exhibited such antibodies. Interestingly, antibodies against the HERV-W_486–504_ peptide were only found in 2 out of the 20 MOGAD patients (10%; [CI]: 55–83%) and 2 out of the 20 NMOSD patients (10%; [CI]: 52–78%). Within the MS group, 6 of the positive individuals were female, and 1 was male. These MS patients had an average EDSS score of 2 and a disease duration of 7 ± 4.5 years. 

When considering the subset of EBNA1_386–405_-positive subjects (*n* = 25), 8 out of 13 MS patients (61%) exhibited double positivity for both EBNA1_386–405_ and HERV-W_486–504_ antibodies. Among the MOGAD patients, 2 out of 7 (28%) showed this double positivity, while only one NMOSD patient with AQP4-positivity exhibited it. None of the HCs displayed double positivity for these viral peptides. These findings suggest a potential connection between EBV infection and HERV-W activation in certain MS subjects, and possibly in some MOGAD patients. However, it is worth noting that, based on our data, further confirmation is required to establish a potential association between these viruses and NMOSD patients, regardless of AQP4 status.

## 3. Discussion

In our study, we detected significantly elevated levels of antibodies against EBV EBNA1 in patients with relapsing–remitting MS and MOGAD. Importantly, this phenomenon was absent in patients with NMOSD. This finding adds to the growing body of evidence supporting the role of EBV in the pathogenesis of MS and potentially in MOGAD, even in the Japanese population. Additionally, we made an intriguing observation—a robust correlation between anti-EBV and anti-HERV-W antibodies in MS patients. This points to a potential synergy between these two viruses in influencing the course of the disease.

Prior studies have already reported the presence of both EBV and HERV-W in MS patients across different countries [30,31]. However, these studies predominantly focused on the RNA expression of HERV-W, overlooking the humoral response against it. Unlike NMOSD and MOGAD, which exhibit clear antibody associations, MS has traditionally been seen as a T-cell-driven disease. Nevertheless, recent developments in B-cell-targeted therapies have stimulated interest in exploring the regulatory and antigen-presenting roles of B cells [32]. This emphasizes the dysregulation of both cell-mediated and humoral components of the adaptive immune system in these disorders.

Regarding MS, the simultaneous presence of EBV and HERV-W in relapsing–remitting MS patients indicates that EBV infection may trigger the expression of specific HERV-W proteins, possibly exacerbating the disease [18]. It is important to note that identifying the transition from relapsing to progressive symptoms in MS, which occurs in about 80% of patients [4], remains challenging. One hypothesis arising from our findings is that EBV may act as a disease trigger, while HERV-W activation could be linked to disease progression in specific susceptible individuals. Thus, it is of interest to conduct seroprevalence studies in patients with progressive forms of MS to identify biomarkers that can predict or differentiate between different disease courses. EBV infection precedes the diagnosis of demyelinating disorders; however, the timing and nature of the relationship between EBV infection and demyelinating disorders vary among individuals, and the exact mechanisms are not fully understood. Some studies suggest a possible link between becoming infected with EBV and an increased likelihood of developing MS, particularly for those who had mononucleosis during adolescence or young adulthood [33]. Interestingly, even though relapsing–remitting MS involves focal inflammation, many people with MS experience disability progression independent of relapse activity (PIRA) [34]. Among the various factors contributing to delayed neurodegenerative processes associated with late-onset disability progression, viral infections seem to be potential drivers. An alternative hypothesis is that, in progressive forms of MS, infections such as EBV may temporarily exacerbate pre-existing symptoms. For instance, infections might activate HERV-W, leading to the overexpression of envelope proteins with proinflammatory effects. In addition to initiating MS, EBV and HERV-W might also play a role in the more advanced stages of the disease.

In the context of MOGAD, there have been a few case reports linking the disease to viral infections such as influenza and EBV [35]. However, the precise pathophysiological mechanism of post-infectious autoimmunity in MOGAD remains unclear. Notably, anti-MOG antibodies are detected in various demyelinating diseases, including AQP4-seronegative NMOSD, recurrent optic neuritis, and acute disseminated encephalomyelitis (ADEM) [36]. In one case, adult-onset ADEM was associated with EBV infection, raising questions about the direct infiltration of the virus into nervous tissue or secondary immunological mechanisms triggered by viral infection as potential underlying factors in MOGAD-related diseases [37]. Even though we detected a significant presence of anti-EBNA1 antibodies in the serum of MOGAD patients, it is crucial to investigate the presence of these viruses in CSF samples to assess their potential to cause neuroinfections in future studies.

In NMOSD, a variety of infectious agents have been suggested as potential triggers, but the evidence for these associations remains incomplete [38]. In our investigation, we did not find high antibody titers against EBV or HERV-W in NMOSD patients. This may be due, in part, to the relatively small sample size. Additionally, in NMOSD patients, the humoral response against EBV appears to be directed against different targets. Notably, elevated anti-early antigen (EA) IgG antibodies were observed in NMOSD patients, indicating recent reactivation of EBV infection [20]. Conversely, the presence of EBNA1 antibodies, which are produced late in the course of infection, suggests prior exposure to the virus [39].

It is plausible that persistent and active EBV replication may play a role in the pathogenesis of NMOSD. The absence of antibodies against HERV-W in AQP4-positive NMOSD patients further supports the hypothesis that HERV-W activation may be linked to EBV. An intriguing point to note is that a substantial portion of NMOSD patients—about 40%—are double seronegative for both AQP4 and MOG antibodies or have an unknown AQP4-IgG status [40]. This subgroup warrants special attention, as the presence of EBV and HERV-W might play a more direct role in their autoimmune processes, potentially through mechanisms like molecular mimicry. These seronegative NMOSD patients could have specific genetic backgrounds that make them more susceptible to developing humoral autoimmunity.

Our study has several limitations. Firstly, it is retrospective. Secondly, the sample size for NMOSD and MOGAD patients was relatively small. Therefore, further studies should involve a larger cohort and include the study of paired sera and CSF samples. Furthermore, the examination of different antigenic components of these viruses is warranted. In addition, it is of utmost significance to evaluate T-cell reactions to these peptides and their potential influence on the regulation of neuroinflammation. This could be accomplished by utilizing animal models, such as active and passive EAE.

In conclusion, our research has provided valuable insights into the complex relationships between EBV, HERV-W, and different autoimmune demyelinating diseases in Japan. While these findings raise intriguing questions, there is a great deal more to investigate and comprehend in this continuously advancing field of research.

Our data support the rationale for investigating the potential therapeutic effects of antiviral agents in MS. This is particularly crucial as anti-inflammatory disease-modifying therapies alone prove inadequate in mitigating disability progression in secondary progressive MS. An integrated approach, involving combined therapies to target factors that could potentially hasten MS-related neurodegeneration, should be considered.

## 4. Materials and Methods

### 4.1. Patients

The retrospective analysis involved a cohort of 116 Japanese participants aged over 18 who were recruited from the Juntendo University School of Medicine in Tokyo, Japan. This research received ethical approval from the Juntendo University School of Medicine’s ethics committee (Approval No: 205) and adhered to the ethical guidelines set forth by the World Medical Association in the Declaration of Helsinki. All study participants provided written informed consent prior to their inclusion in the research.

The study included three distinct patient groups. The first group consisted of 34 individuals diagnosed with relapsing–remitting MS, with a gender distribution of 26 females and 8 males, and an average age of 40 years (±9). The diagnosis of RRMS was made by a neurologist following the 2017 McDonald criteria [41]. These patients had an average disease duration of 7 years (±6), with the onset of MS occurring at an average age of 32 (±8). At their last visit, the mean EDSS score for these individuals was 2 (±2).

The second group, comprising 20 subjects, was diagnosed with NMOSD. Within this group, there were 15 females and 5 males, with a mean age of 45 (±16). Diagnosis was in accordance with the 2015 international consensus diagnostic criteria [42], and all NMOSD patients had either recurrent optic neuritis, longitudinally extensive transverse myelitis, or both, and 12 out of 20 (60%) were AQP4-positive. The mean disease duration for NMOSD patients was 8 years (±7), and the average age at onset was 43 (±14). The EDSS score for this group was 4 (±2).

A third group included 20 patients with MOGAD. Within this group, there were 14 females and 6 males, with a mean age of 39 (±10). Diagnosis of MOGAD was based on international diagnostic criteria for MOGAD [43], and all patients were positive for MOG antibodies. These patients had a disease duration of 6 years (±4), with the onset typically occurring at an average age of 32 (±10). The EDSS score for MOGAD patients was 3 (±3).

None of the patients included in this study had received any treatment for their respective conditions for at least 3 months before serum sample collection. To establish baseline data, a control group was established, consisting of 40 individuals matched for age and gender (30 females and 10 males, mean age 44 ± 15) who did not exhibit any specific neurological disorders. Table 1 provides a summary of the baseline characteristics of the study participants.

### 4.2. Synthetic Peptides

Peptides EBNA1_386–405_ (sequence CSQSSSSGSPPRRPPPGRRPF) derived from the EBNA1 protein (UniProt accession number P03211), and HERV-W_486–504_ (sequence CQIVLQMEPQMQSMTKIYRG) originating from the recombinant envelope protein (UniProt accession number Q991W9), were chemically synthesized to a purity exceeding 95% by Synpeptide Co., Shanghai, China.

The EBNA1_386–405_ epitope is located between the protein’s N-terminal and its DNA-binding domain. Research has provided evidence of molecular mimicry between EBNA1_386–405_ and GlialCAM, a glial cell adhesion molecule expressed in the CNS [44].

The HERV-W_486–504_ epitope represents a preserved segment within the HERV-W protein’s envelope sequences. Extracellular RNA sequences from this protein have been identified in the blood and cerebrospinal fluid (CSF) of individuals with MS [29].

### 4.3. Peptide-Based Indirect Enzyme-Linked Immunosorbent Assays (ELISA) 

To perform the peptide-based indirect ELISA, we employed the Imject maleimide-activated bovine serum albumin (BSA) spin kit from Thermo Fisher Scientific, Waltham, MA, USA, as previously published [19]. This kit was selected to ensure that antigenic epitopes remain accessible for antibody binding, preventing any masking effect. The procedure consisted of activating the BSA carrier protein with reactive sulfhydryl maleimide, purifying it, and then crosslinking it with the sulfidyl group (-SH) moiety within the cysteine-containing peptides EBNA1_386–405_ and HERV-W_486–504_, following the manufacturer’s instructions.

In brief, to determine the optimal coating conditions, we conducted titration experiments. Nunc-immuno MicroWell 96-well solid plates from Thermo Fisher Scientific, Waltham, MA, USA, were coated with 50 µL per well of the EBNA1_386–405_ or HERV-W_486–504_ peptides, which were diluted in ELISA coating buffer from Bio-Rad, Tokyo, Japan, to achieve a final concentration of 10 μg/mL. The plates were incubated overnight at 4 °C. Subsequently, the microplate was blocked by adding 200 µL per well of Blocking One from Nakalai Tesque, Kyoto, Japan, and this was performed for 1 h at room temperature.

Sera samples were then added to the duplicate wells, with a 1:100 dilution in Blocking One, and incubated for 2 h at room temperature (25 °C). After this incubation, the plates underwent four washes with phosphate-buffered saline with 0.05% Tween 20 (PBS-T). The plates were then incubated with 100 µL per well of horseradish peroxidase-labeled goat anti-human total IgG from Southern Biotech Associates, Inc., Birmingham, AL, USA, for 1 h at room temperature. Following this incubation, the microplates were washed again, and the wells were subjected to an incubation of 100 µL per well of the ABTS Peroxidase System from SeraCare Life Sciences, KPL, Gaithersburg, MD, USA, for 10 min at room temperature in the dark.

The optical density (OD) was measured at 650 nm using a Benchmark Plus Microplate Reader from Bio-Rad, Tokyo, Japan. Wells coated with BSA were included as a negative control, and the mean value obtained from these wells was subtracted from all other data points. The results were normalized against a positive control serum, which was included in all experiments.

### 4.4. Epstein–Barr Virus EBNA1 ELISA

To compare the results obtained from our in-house peptide-based ELISA, we conducted a control ELISA using a commercially available Epstein–Barr Virus EBNA1 ELISA Kit (Abnova, Walnut, CA, USA). This system utilizes a solid phase immunoanalytical test in which a specific recombinant antigen of EBNA1 EBV is bound to the surface of the wells. If relevant antibodies are present in the test sample, they will bind to these immobilized antigens. The test kit includes built-in negative controls and standards for quantitative determination. The human control sera and standards used in the kit were screened for the absence of HBsAg, HCV, and anti-HIV-1,2 antibodies.

In brief, serum samples, controls, and standards were added in duplicate to the wells and subsequently incubated for 30 min at 37 °C. Following this incubation, the plates underwent four washes to remove unbound substances. The plates were then subjected to a 30-min incubation at 37 °C with 200 µL per well of horseradish peroxidase-labeled anti-human IgG antibodies. After this incubation, the microplates were washed again, and the wells were exposed to 100 µL per well of the chromogenic substrate TMB for 15 min in the dark at room temperature. The reaction was halted by adding 100 µL per well of a stop solution, after which absorbance was measured at 450 nm (with a reference reading at 620–690 nm) using a Benchmark Plus Microplate Reader from Bio-Rad, Tokyo, Japan.

As per the manufacturer’s instructions, for a qualitative interpretation of results, the cut-off value was determined by multiplying the mean OD value of the standard by a correction factor specified in the quality control certificate. Samples with OD values below 90% of the cut-off were classified as negative, whereas samples with OD values exceeding 100% of the cut-off were considered positive.

For quantitative interpretation of results, we calculated the antibody titers in arbitrary units per milliliter (AU/mL) by constructing a calibration curve. This curve was generated by plotting the concentration units of the standards on the x-axis against their corresponding absorbance values on the y-axis. We then positioned the absorbance values of the serum samples on the calibration curve to determine their corresponding antibody titer values (AU/mL) on the X-axis. This process allowed for us to quantify the antibody concentration in the tested samples.

### 4.5. Statistics

We conducted statistical analysis employing GraphPad Prism 10.1 software (GraphPad Software, La Jolla, CA, USA). The comparison of ELISA results between patients and HCs was carried out using the non-parametric Mann–Whitney’s U-test. To validate the generated information, we performed a correlation analysis between the anti- EBNA1_386–405_ antibody titers detected via our peptide-based ELISA and those from a commercially available EBNA1 ELISA kit, utilizing the Spearman correlation test. Linear regression analysis was also conducted to examine the correlation between anti- EBNA1_386–405_ and HERV-W_486–504_ antibodies. For assessing the diagnostic accuracy of the ELISA and determining the positivity threshold with a specificity of 95%, we conducted a Receiver Operating Characteristic (ROC) analysis. Statistical significance was defined as a *p*-value less than 0.05.

## Figures and Tables

**Table 1:** Baseline characteristics of Japanese cohort.

	MS (34)	NMOSD (20)	MOGAD (20)	HCs (40)
Gender female/male	26/8	15/5	14/6	30/10
Age (years) mean ± SD	40 ± 9	45 ± 16	39 ± 10	44 ± 15
Age at onset (years) mean ± SD	32 ± 8	43 ± 14	32 ± 10	0
Disease duration (years) mean ± SD	7 ± 6	8 ± 7	6 ± 4	0
EDSS score mean ± SD	2 ± 2	4 ± 2	3 ± 3	0
Anti-AQP4 IgG (% positive)	0	60	0	0
Anti-MOG IgG (% positive)	0	0	100	0

## Data Availability

The data underlying this article will be shared on reasonable request to the corresponding author.
